# The genetic spectrum of congenital ocular motor apraxia type Cogan: an observational study, continued

**DOI:** 10.1186/s13023-023-02706-5

**Published:** 2023-05-02

**Authors:** Simone Schröder, Gökhan Yigit, Yun Li, Janine Altmüller, Hans-Martin Büttel, Barbara Fiedler, Christoph Kretzschmar, Peter Nürnberg, Jürgen Seeger, Valentina Serpieri, Enza Maria Valente, Bernd Wollnik, Eugen Boltshauser, Knut Brockmann

**Affiliations:** 1 Interdisciplinary Pediatric Center for Children with Developmental Disabilities and Severe Chronic Disorders, Department of Pediatrics and Adolescent Medicine, University Medical Center, Göttingen, Germany; 2 Institute of Human Genetics, University Medical Center, Göttingen, Germany; 3 Cologne Center for Genomics (CCG) and Center for Molecular Medicine Cologne (CMMC), Faculty of Medicine and University Hospital Cologne, University of Cologne, Cologne, Germany; 4 MVZ Genetikum GmbH, Stuttgart, Germany; 5 Division of Neuropediatrics, Department of General Pediatrics, University Hospital Münster, Münster, Germany; 6 Sozialpädiatrisches Zentrum, Städtisches Klinikum Dresden, Dresden, Germany; 7 Center of Developmental Neurology (SPZ Frankfurt Mitte), Frankfurt, Germany; 8 Neurogenetics Research Center, IRCCS Mondino Foundation, Pavia, Italy; 9 Department of Molecular Medicine, University of Pavia, Pavia, Italy; 10 Neurogenetics Research Center, IRCCS Mondino Foundation, Pavia, Italy; 11 Institute of Human Genetics, University Medical Center, Göttingen, Germany; 12 Cluster of Excellence “Multiscale Bioimaging: from Molecular Machines to Networks of Excitable Cells” (MBExC), University of Göttingen, Göttingen, Germany; 13 Department of Pediatric Neurology (Emeritus), University Children’s Hospital, Zurich, Switzerland; 14 Present Address: Berlin Institute of Health at Charité - Universitätsmedizin Berlin, Core Facility Genomics, Berlin, Germany; 15 Present Address: Max Delbrück Center for Molecular Medicine in the Helmholtz Association (MDC), Berlin, Germany

**Keywords:** Congenital ocular motor apraxia, Cogan syndrome, Joubert syndrome, Molar tooth sign, Ciliopathy, Poretti–Boltshauser syndrome

## Abstract

**Background:**

The term congenital ocular motor apraxia (COMA), coined by Cogan in 1952, designates the incapacity to initiate voluntary eye movements performing rapid gaze shift, so called saccades. While regarded as a nosological entity by some authors, there is growing evidence that COMA designates merely a neurological symptom with etiologic heterogeneity. In 2016, we reported an observational study in a cohort of 21 patients diagnosed as having COMA. Thorough re-evaluation of the neuroimaging features of these 21 subjects revealed a previously not recognized molar tooth sign (MTS) in 11 of them, thus leading to a diagnostic reassignment as Joubert syndrome (JBTS). Specific MRI features in two further individuals indicated a Poretti–Boltshauser syndrome (PTBHS) and a tubulinopathy. In eight patients, a more precise diagnosis was not achieved. We pursued this cohort aiming at clarification of the definite genetic basis of COMA in each patient.

**Results:**

Using a candidate gene approach, molecular genetic panels or exome sequencing, we detected causative molecular genetic variants in 17 of 21 patients with COMA. In nine of those 11 subjects diagnosed with JBTS due to newly recognized MTS on neuroimaging, we found pathogenic mutations in five different genes known to be associated with JBTS, including *KIAA0586, NPHP1, CC2D2A, MKS1*, and *TMEM67*. In two individuals without MTS on MRI, pathogenic variants were detected in *NPHP1* and *KIAA0586*, arriving at a diagnosis of JBTS type 4 and 23, respectively. Three patients carried heterozygous truncating variants in *SUFU*, representing the first description of a newly identified *forme fruste* of JBTS. The clinical diagnoses of PTBHS and tubulinopathy were confirmed by detection of causative variants in *LAMA1* and *TUBA1A*, respectively. In one patient with normal MRI, biallelic pathogenic variants in *ATM* indicated variant ataxia telangiectasia. Exome sequencing failed to reveal causative genetic variants in the remaining four subjects, two of them with clear MTS on MRI.

**Conclusions:**

Our findings indicate marked etiologic heterogeneity in COMA with detection of causative mutations in 81% (17/21) in our cohort and nine different genes being affected, mostly genes associated with JBTS. We provide a diagnostic algorithm for COMA.

**Supplementary Information:**

The online version contains supplementary material available at 10.1186/s13023-023-02706-5.

## Background

When David C. Cogan in a *Jackson Memorial Lecture* in 1952 described four children with a distinct disturbance of voluntary horizontal gaze characterized by the “inability to turn the eyes voluntarily in a direction for which there is full involuntary … control” accompanied by compensatory, jerky head movements, he coined the term *congenital ocular motor apraxia* (COMA) [1]. Ocular apraxia designates the incapacity to initiate eye movements performing rapid gaze shift, so called saccades. In most patients, COMA affects horizontal, occasionally also vertical saccades.

In 2016 we reported an observational study aiming at the nosological delineation of congenital ocular motor apraxia type Cogan [2]. We had recruited a cohort of 21 previously unreported patients (8 female, 13 male, ages at that time ranging from 2 to 24 years) diagnosed as having COMA. Inclusion criteria comprised early-onset OMA (diagnostic recognition within the first year of life), availability of an MRI in technical quality adequate for assessment of especially brainstem and cerebellum, and written informed consent of the parents or the patient or both. Patients with already established diagnosis of Joubert Syndrome (JBTS) based on presence of the molar tooth sign (MTS) on MRI were not included. Re-evaluation of MRI data sets of all 21 subjects disclosed a so far unrecognized molar tooth sign diagnostic for JBTS in 11 patients, neuroimaging features of Poretti-Boltshauser syndrome (PTBHS) in one subject and cerebral malformation suspicious of a tubulinopathy in another individual. MRI revealed hypo-/dysplasia of the vermis cerebelli in four and no abnormalities in the remaining four patients. These results provided strong evidence for the notion, that COMA does not designate a nosological entity, but rather a neurological symptom with heterogeneous etiology [2].

Pursuing this cohort of 21 patients with COMA, we aimed at clarification of the definite genetic etiology in all subjects. Here, we report the molecular genetic findings in 17 individuals with conclusive genetic diagnoses.

## Methods

This study was approved by the ethics committee of the University Medical Center, Göttingen, Germany (file no. 19/5/14). All participating families provided written informed consent.

### Patient cohort

Recruitment of the 21 patients with COMA was described previously [2]. Briefly, an email based acquisition of rare neurological disorders in childhood [3] was used to collect subjects with early-onset OMA (diagnostic recognition within the first year of life), who had received MRI in technical quality adequate for assessment of brainstem and cerebellum in particular.

### Genetic testing

The genetic findings reported here were obtained partly in the setting of patient care and partly within the frame of research projects. For most of those 11 patients who were diagnosed as having JBTS based on recognition of the MTS in re-evaluation of their MRI, established diagnostic NGS pathways as part of patient care were used including molecular genetic panels or whole exome sequencing (WES).

One subject (#7) with neuroimaging features indicating PTBHS was included in a research project aiming at clinical, neuroradiological and molecular characterization of this condition [4].

The individual (#20) with MRI findings pointing to a tubulinopathy received genetic testing in a candidate gene approach.

In two subjects (#8 and #9) with newly recognized JBTS and in the remaining eight subjects (#3, #6, #10, #11, #12, #14, #18, and #19) with inconclusive MRI findings, molecular genetic investigations were performed as part of this research project.

Methods of WES and variant screening were reported previously [5, 6].

In patients #8, #9, and #10, trio-based WES of the affected subjects and their parents was carried out using the NimbleGen SeqCap EZ Human Exome Library v2.0 enrichment kit (Roche) on an Illumina HiSeq4000 sequencer. In patients #14 and #19, trio-based WES of the affected subjects and their parents was carried out using the Agilent SureSelect V6 (Agilent) on an Illumina HiSeq4000. WES data analysis and filtering of mapped target sequences was performed using the ‘Varbank’ exome analysis pipeline of the Cologne Center for Genomics (CCG, University of Cologne, Germany), and data were filtered for high-quality (coverage of more than 6 reads, a minimum quality score of 10), rare (minor allele frequency, MAF < 1.0%) variants. Identified variants were confirmed by PCR amplification as well as subsequent Sanger sequencing and tested for cosegregation within the respective families.

Additional file 1: Table S1 provides details of molecular genetic tests performed in each patient.

## Results

Table 1 displays the genetic findings in 17 of 21 patients with COMA together with their main clinical and neuroimaging features. Numbering of the patients was adopted from ref. [2]. In nine of those 11 subjects diagnosed with JBTS due to newly recognized MTS on neuroimaging, we found pathogenic mutations in five different genes known to be associated with JBTS.

**Table 1:** Clinical, neuroimaging and genetic features of 21 patients with "congenital ocular motor apraxia type Cogan" (COMA)

Patient # (origin)	Sex	Current age (years)	Development	Neurological findings		MRI features	Mutated gene, pathogenic variants	Diagnostic assignment	References
				Ocular apraxia [age at onset (months)/course/age at disappearance]	Early onset ataxia				
1 (A)	f	10	SD, LD	3/↓/−	Yes	MTS, vermian hypo-/dysplasia	*MKS1* comp. het., c.1115_1117delCCT (p.Ser372del), c.1476T > G (p.Cys492Trp)	JBTS28	
2 (D)	m	13	UWD, SD, LD	8/↓/−	Yes	MTS, superior vermian hypoplasia, slightly enlarged external csf spaces	*CC2D2A* comp. het., c.3289delG (p.Val1097Phefs*2) (possible splice change), c.4583G > A (p.Arg1528His)	JBTS9	
3 (D)	f	18	UWD, CDN	3/↓/−	No	Normal	ES provided no conclusive result	“COMA “	
4 (D)	m	24	UWD, SD, LD	3/↓/−	Yes	MTS, superior vermian dysplasia	*TMEM67* comp. het., c.622A > T (p.Arg208*), c.1634G > A (p.Gly545Glu)	JBTS6	
5 (TR)	f	12	UWD, SD, ID	4/↓/−	Yes	MTS, superior vermian hypo-/dysplasia	ES provided no conclusive result	JBTS	
6 (D)	f	10	UWD, CDN	6/↓/−	Yes	vermian dysplasia, otherwise normal	*KIAA0586* comp. het., c.428delG (p.Arg143Lysfs*4), c.3888delC (p.Ile1297Serfs*19)	JBTS23	
7 (TR)	m	24	UWD, SD, CDN	6/ ↔ /−	Yes	cerebellar cysts, cerebellar hypoplasia, square 4th ventricle	*LAMA1* hom., c.756delT (p.Ile252Metfs*10)	PTBHS	
8* (D)	m	30	UWD, SD, ID	6/↓/4 years	Yes	MTS, vermian hypo-/dysplasia	*KIAA0586* comp. het., c.428delG (p.Arg143Lysfs*4), c.1413-1G > C (splice site variant)	JBTS23	[5]
9* (D)	f	27	SD, LD	11/↓/5 years	Yes	MTS, otherwise normal	*KIAA0586* comp. het., c.428delG (p.Arg143Lysfs*4), c.1413-1G > C (splice site variant)	JBTS23	[5]
10 (T)	m	15	UWD, SD, LD	6/↓/−	Yes	SCP mildly horizontalized, elongated, thickened, inferior vermian dysplasia, upper vermis split	*SUFU* het., c.83C > A (p.Ser28*)	*forme fruste* of JBTS	[6]
11 (D)	m	22	UWD, SD, CDN	2/↓/−	No	Normal	*NPHP1* hom. deletion of whole gene	JBTS4	
12 (D)	f	13	UWD, SD	10/↓/−	Yes	Normal	ES provided no conclusive result	“COMA”	
13 (D/UK)	m	9	UWD, CDN	8/↓/−	Yes	MTS, otherwise normal (mild superior vermian hypo-/dysplasia??)	*KIAA0586* comp. het., c.428delG (p.Arg143Lysfs*4), deletion of exons 8, 9 and 10	JBTS23	
14 (CH)	m	13	UWD, SD, CDN	6/↓/−	Yes	SCP mildly horizontalized, elongated, thickened, mild vermis folia dysplasia, upper vermis split	*SUFU* het., c.1099G > T (p.Glu367*)	*forme fruste* of JBTS	[6]
15 (D)	m	12	UWD, SD, ID	8/↓/−	Yes	MTS, superior vermian dysplasia	*NPHP1* hom. deletion of whole gene	JBTS4	
16* (D)	m	28	UWD, SD, CDN	6/↓/−	Yes	MTS, vermian hypo-/dysplasia	*KIAA0586* comp. het., c.428delG (p.Arg143Lysfs*4), deletion of exons 8, 9 and 10	JBTS23	
17* (D)	m	24	UWD, SD, CDN	3/↓/−	Yes	MTS, superior vermian hypo-/dysplasia	*KIAA0586* comp. het., c.428delG (p.Arg143Lysfs*4), deletion of exons 8, 9 and 10	JBTS23	
18 (D)	f	29	UWD, LD	4/↓/−	Yes	Normal	*ATM* comp. het., c.1066-6T > G (p.?), c.2250G > A (p.Ile709_Lys750del)	variant A-T	[7]
19 (D)	f	12	normal	6/↓/−	No	SCP mildly horizontalized, elongated, thickened, superior vermian dysplasia, upper vermis split	*SUFU* het, c.479delA (p.His160Leufs*20) de novo	*forme fruste* of JBTS	[6]
20 (R/K)	m	17	UWD, SD, ID	5/↓/−	Yes	Enlarged ventricles, dysmorphic basal ganglia, hypoplastic corpus callosum, abnormal proportions of brainstem, suspicious of tubulinopathy	*TUBA1A* het., c.82C > T (p.His28Tyr), de novo	TUBA1A-associated brain malformation	
21 (D)	m	12	UWD, SD, LD	8/↓/−	Yes	MTS, callosal agenesis, vermian hypo-/dysplasia, hippocampal malrotation, dysplastic tectal plate	*RPGRIP1L* two het. variants on the same (maternal) allele, likely not causative: c.171G > T (p.Leu57Phe), c.628A > G (p.Asn210Asp)	JBTS	

^*^ = #8 and #9 as well as #16 and #17 are siblings; A = Albanian origin; CH = Swiss origin; D = German origin; K = Kazakh origin; R = Russian origin; T = Turkish origin; UK = British origin; m = male; f = female; UWD = unaided walking delayed; SD = speech delay; ID = intellectual disability; LD = learning disability; CDN = cognitive development normal; ↓ = attenuating; ↔  = unchanged; MTS = molar tooth sign; A-T = ataxia-telangiectasia; JBTS = Joubert syndrome; PTBHS = Poretti-Boltshauser syndrome; het. = heterozygous; hom. = homozygous; SCP = superior cerebellar peduncles

Adopted and modified from Wente et al. reference [2]

Six individuals [#8, #9, and #16, #17 (two pairs of siblings), #6, and #13] carried biallelic pathogenic variants in the *KIAA0586* gene associated with JBTS23.

Heterozygous truncating pathogenic causative variants in the *SUFU* gene were detected in three subjects, as reported previously in detail [6].

In two subjects (#11, #15), a homozygous deletion of the whole *NPHP1* gene was found, leading to a diagnosis of JBTS4. Of note, in patient #11 our in-depth re-evaluation of the neuroimaging data sets did not reveal a MTS, and the diagnosis of JBTS4 was based only on the molecular genetic finding.

Biallelic pathogenic variants in the genes *MKS1*, *CC2D2A*, and *TMEM67* were found in one patient each (#1, #2, #4), leading to a diagnosis of JBTS28, JBTS9, and JBTS6, respectively.

The diagnosis of Poretti–Boltshauser syndrome in subject #7 was based on the characteristic neuroimaging features and now confirmed by identification of a homozygous pathogenic variant in the *LAMA1* gene.

In one individual (#20) with MRI findings pointing to a tubulinopathy, genetic testing revealed a heterozygous de novo likely pathogenic variant in the *TUBA1A* gene.

In one female patient (#18) in this cohort, biallelic variants in the *ATM* gene were detected, proven to be pathogenic in a separate research project, leading to the diagnosis of variant ataxia-telangiectasia [7].

Molecular genetic findings in the siblings #8 and #9 [5], in subjects #10, #14, and #19 [6] as well as in patient #18 [7] were reported previously, as these genetic results implicated specific new insights related with the respective conditions.

In patient #21, neuroimaging had shown the rare combination of MTS with agenesis of the corpus callosum (ACC). ES revealed two heterozygous variants in the *RPGRIP1L* gene, which were located on the same (maternal) allele, however. Biallelic pathogenic variants in *RPGRIP1L* are associated with JBTS7 [8]. Moreover, mutations in *RPGRIP1L* have been linked with ACC [9–11]. Reverse phenotyping including laboratory investigations of renal function, renal ultrasound, and funduscopy at age 12 years did not provide any evidence for nephronophthisis or retinal dystrophy, known to be part of the clinical spectrum of JBTS7*.* Taken together, we did not gather sufficient evidence for pathogenicity of these two monoallelic variants in *RPGRIP1L*.

In three patients (#3, #5, #12), WES did not provide conclusive results, and the etiology of COMA remains unsolved in these cases. Among them, patient #5 had a clear MTS on neuroimaging, thus arriving at a clinical diagnosis of JBTS, however without clarification of the genetic basis.

Table 2 lists all mutated genes and the number off affected subjects in our cohort.

**Table 2:** Mutated genes in 17 patients with COMA

Mutated gene	Clinical condition	Inheritance	OMIM #	Number of patients in this cohort
*KIAA0586*	JBTS23	AR	616490	6
*SUFU*	*forme fruste* of JBTS	AD	n.a.	3
*NPHP1*	JBTS4	AR	609583	2
*ATM*	Variant A-T	AR	208900	1
*CC2D2A*	JBTS9	AR	612285	1
*LAMA1*	PTBHS	AR	615960	1
*MKS1*	JBTS28	AR	617121	1
*TMEM67*	JBTS6	AR	610688	1
*TUBA1A*	*TUBA1A*-associated brain malformation	AD	611603	1

*AD* autosomal dominant, *AR* autosomal recessive, *A-T* ataxia-telangiectasia, *JBTS* Joubert syndrome, *PTBHS* Poretti-Boltshauser syndrome

## Discussion

In this cohort of 21 patients with COMA, molecular genetic investigations including WES, panel, or single gene analyses revealed causative genetic findings in 17 subjects. The clinical and neuroimaging diagnosis of JBTS established in 11 subjects was confirmed in nine patients by causative molecular genetic findings. In two individuals with clear MTS on neuroimaging no conclusive genetic result was obtained, using WES. Two other patients without definite MTS arrived at a genetic diagnosis of JBTS4 and JBTS23 due to pathogenic variants in the *NPHP1* and *KIAA0586* gene, respectively. This observation sheds new light on the doctrinal statement, that the neuroimaging feature of MTS is a prerequisite for the diagnosis of JBTS. In this study we found both, subjects with and without definite MTS to carry pathogenic biallelic mutations in *NPHP1* or *KIAA0586*, two *bona fide* JBTS genes. Apart from the methodological issues which might compromise recognition of the MTS (technically appropriate MRI, expert assessment), increasing evidence indicates that the ciliopathic midbrain-hindbrain malformations do not allow for a clear-cut separation between MTS and normal. Instead, they rather shape a spectrum of anomalies with smooth transitions between normal and strikingly abnormal morphology of brainstem and cerebellum, as exemplary observed in *SUFU*-associated conditions [6, 12]. However, we would like to emphasize, that, as a rule, in JBTS the large majority of patients have a typical MTS; neuroimaging features resembling a mild MTS (as discussed below with *SUFU*-associated conditions) are occasionally observed, and normal MRI is exceptionally rare.

In three patients with mild cerebellar abnormalities including prominent, thickened, elongated superior cerebellar peduncles, vermis folia dysplasia and upper vermis split, but without definite MTS on MRI, we detected heterozygous truncating mutations in the *SUFU* gene. Together with further patients recruited subsequently, these subjects represent the first examples of a newly identified *forme fruste* of JBTS [6]. More recently, additional 22 patients with *SUFU* haploinsufficiency and a neurodevelopmental phenotype at the mild end of the Joubert syndrome spectrum were reported [12]. All these patients had persistent COMA.

Among those 11 subjects of our cohort, who carried causative mutations associated with any of the subtypes of JBTS, *KIAA0586* was the gene most frequently affected. Although *KIAA0586* was recognized to be one of the most prevalent six JBTS genes [13], the prevalence of *KIAA0586*-associated JBTS23 among all JBTS subtypes was estimated to be 2.5% [14], 5% [15] and 7% [13] in large cohorts of JBTS patients reported previously. In this respect, the high prevalence of 19% (6 individuals from 4 out of 21 families) in our COMA cohort is surprising. We cannot explain why *KIAA0586* is prevailing in our cohort and presume that this is likely a coincidental finding. While some of the abovementioned previous studies provided information on the prevalence of retinal dystrophy and coloboma in the different subtypes of JBTS, no such date are available for COMA.

Of note, in one subject (#18) with recognition of COMA as early as 4 months of age we identified two variants in the *ATM* gene in compound heterozygous state [7]. While the pathogenicity of the splice-donor site variant c.2250G > A, resulting in skipping of exon 14, was already convincingly documented, it was a matter of longstanding debate whether the 2nd variant, the splice-acceptor site variant c.1066-6T > G, is disease-causing. This variant leads to “leaky” splicing of exon 11 and thus to exon skipping, thereby to frameshift and premature protein truncation. In a separate study we showed that these two variants in compound heterozygous state lead to reduced expression of ATM protein and residual activity of the ATM kinase at a level consistent with variant ataxia-telangiectasia (A-T) [7]. To our knowledge, this is the first report of ocular motor apraxia as the initial symptom of A-T.

Patient #7 showed characteristic features of Poretti-Boltshauser syndrome (PTBHS) on neuroimaging, and detection of a homozygous pathogenic variant in *LAMA1* confirmed this diagnosis. Ocular motor apraxia was reported to be part of the clinical phenotype in PBS already in the first description [16] as well as in subsequent publications [4] and is listed in the Clinical Synopsis of PTBHS in OMIM.

Although the number of patients with COMA gathered in this cohort is small, the genetic findings reported here depict a somewhat representative spectrum of genetic etiologies in COMA. When we recruited our cohort of patients diagnosed as having COMA, we excluded all subjects with a pre-existing diagnosis of JBTS, based on recognition of the MTS in neuroimaging [2]. Thus, our cohort has a clear bias towards diverse rare causes of COMA, others than JBTS. However, the genetically heterogeneous group of JBTS accounts by far for the most cases of COMA [17].

OMIM currently lists 40 different genetically defined subtypes of JBTS. For 27 of these subtypes, ocular apraxia is specified as a clinical feature in the Clinical Synopsis of the respective JBTS subtype in OMIM. For further four JBTS subtypes, a PubMed search retrieves reports of ocular apraxia as part of the clinical phenotype (type 9 [18]; type 23 [5, 15] and this report; type 26 [19], and type 30 [20]). In addition, a recent review of ocular manifestations of JBTS [21] listed ocular apraxia as a clinical feature in further two subtypes of JBTS, namely types 10 and 13, associated with *OFD1* and *TCTN1*.

For the remaining seven subtypes of JBTS, a PubMed search using the term “ocular apraxia” and the name of the respective gene does not detect any hits. However, when novel associations of a JBTS phenotype with a particular gene were reported, some of these papers strongly focus on the molecular genetic results and other laboratory tests. The clinical features were mentioned more in passing in these reports. Furthermore, several of these genetic reports described only single families. Occasionally, “abnormal eye movements” are listed among the clinical features, which may include ocular apraxia. Thus, ocular apraxia might occur in additional subtypes of JBTS [22].

In a textbook of neuroophthalmology published in 2010 [23], COMA was ascribed to three major clinical conditions:

1. A benign (“idiopathic”) variant with normal findings in both, neurological examination and neuroimaging, but occasional muscular hypotonia, motor and speech delay, as well as ataxia;
2. A variant with non-progressive, “non-inherited” structural brain anomaly due to either a developmental aberration or acquired lesion, e.g., dysgenesis of the cerebellar vermis or corpus callosum, Dandy–Walker malformation, gray matter heterotopias, or hypoxic-ischemic encephalopathy; and
3. A spectrum of genetic multisystem disorders including JBTS, Jeune syndrome, and a subset of patients with Leber congenital amaurosis [23].

Our observations reported here together with evidence emerging over the past decade indicate that the borders of these three categories are blurring. E. g., the “benign, idiopathic” variant (1) with muscular hypotonia, mild developmental delay, and ataxia fits well with the *forme fruste* of JBTS associated with heterozygous truncating *SUFU* variants [6, 12]. These patients do show nonprogressive structural brain anomalies (2) comprising vermis folia dysplasia and upper vermis split discernible on neuroimaging, and there is a smooth transition to definite JBTS (3) with a full-blown MTS.

Taken together, our results show that COMA presents a neurological symptom with marked etiologic heterogeneity. Genetic causes by far outnumber acquired conditions, and among the inherited ailments, the genetically heterogeneous group of Joubert syndromes account for the vast majority of OMA with onset in infancy.

Based on these data we propose the following recommendations for the diagnostic approach in patients with COMA: Once the symptom of early-onset ocular motor apraxia is assured by a pediatric neurologist or neuroophthalmologist or both, further diagnostic steps should be initiated, if not already performed. Regional and local availabilities may shape the sequence of these investigations. Advisable are

1. a thorough physical examination with focus on congenital anomalies, e.g. polydactyly, thoracic dysplasia, other skeletal dysplasia,
2. an ophthalmological examination with focus on retinal dystrophy and coloboma,
3. MRI of the brain in technical quality (angulation, slice thickness) adequate for assessment of especially brainstem and cerebellum, and review of this MRI by a neuroradiologist or neurologist with experience in pediatric posterior fossa diseases, particularly malformations.

**Fig. 1:**
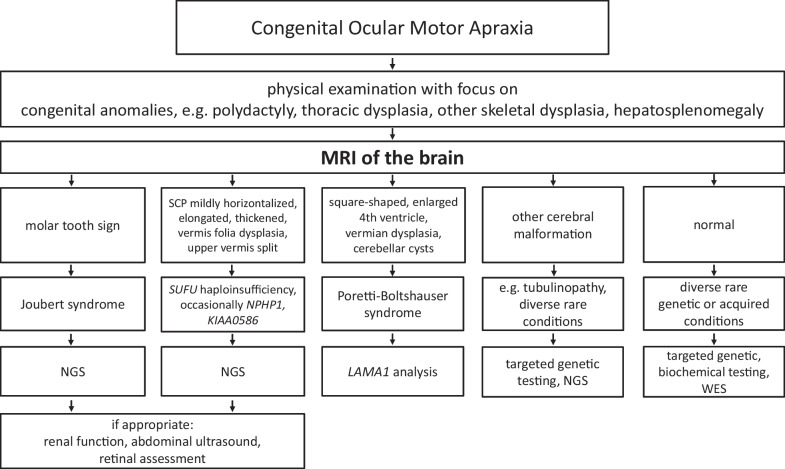
Algorithm for the diagnostic approach in COMA. *NGS* next generation sequencing, *SCP* superior cerebellar peduncles, *WES* whole exome sequencing

Figure 1 provides an algorithm for the diagnostic approach in COMA.

The main diagnostic procedure in patients with early-onset ocular motor apraxia is a molecular genetic investigation using NGS-based approaches. Both, a specific multi-gene panel analysis or an whole exome sequencing (WES) strategy can be performed. The reason why the authors prefer an initial multi-gene panel approach is mainly based on the current fact that variant calling of indels, mid-sized deletion and duplications from NGS data is still often more robust from multi-gene-panels. Moreover, complete coverage of all genes and all coding regions might not be achieved for 100% of the coding regions by WES. However, we also note that WES technology as well as its bioinformatics applications for the detection of structural changes are steadily improving. Applying whole genome sequencing in routine diagnostics in the future certainly will change this strategy allowing high quality sequencing and analysis of all genes.

The results of the MRI will decisively guide the subsequent genetic testing. E.g., neuroimaging features of PTBHS are pathognomonic, and if this pattern is seen on MRI, *LAMA1* analysis can be initiated straightforwardly. Detection of a brain malformation like lissencephaly or consistent with a tubulinopathy will prompt appropriate NGS panel testing, and a JBTS panel would be misleading in these cases.

As discussed above, the numerous subtypes of JBTS account for the vast majority of cases of COMA. Results of the abovementioned examinations might allow for narrowing down the spectrum of genetic conditions associated with COMA in general and thus may help in piloting the evaluation of the molecular genetic findings in a given patient. If molecular genetic testing reveals a JBTS subtype known to be associated with nephronophthisis, investigation of renal function (glomerular filtration rate, urine osmolality value [24]) and renal ultrasound are recommended.

In subjects with inconclusive neuroimaging, laboratory investigations accounting for various very rare causes of COMA (e.g. A-T, Gaucher disease, succinic semialdehyde dehydrogenase deficiency, to name just a few) are advisable. Depending on the local availability, early implementation of WES may shorten the duration of the diagnostic trajectory.

## Conclusions

Our findings indicate marked etiologic heterogeneity in COMA with detection of causative mutations in 81% (17/21) in our cohort and nine different genes being affected, mostly genes associated with JBTS. We provide a diagnostic algorithm for COMA, which may help clinicians in the diagnostic work-up of a patient with early-onset ocular apraxia.

## Supplementary Information

**Additional file 1: Table S1.** Molecular genetic investigations and findings in 21 patients with “congenital ocular motor apraxia type Cogan”.

## Data Availability

Not applicable.

## Abbreviations

A-T: Ataxia-telangiectasia
COMA: Congenital ocular motor apraxia
ES: Exome sequencing
JBTS: Joubert syndrome
MTS: Molar tooth sign
OMA: Ocular motor apraxia
PTBHS: Poretti–Boltshauser syndrome
