# Supplementary material for: The genetic spectrum of congenital ocular motor apraxia type Cogan: an observational study, continued
Source: Orphanet J Rare Dis. 2023 May 2;18:101. doi: 10.1186/s13023-023-02706-5 (PMC10155342; doi:10.1186/s13023-023-02706-5)
Supplement: Supplementary file 1 — Additional file 1: Table S1. Molecular genetic investigations and findings in 21 patients with “congenital ocular motor apraxia type Cogan”. [file 13023_2023_2706_MOESM1_ESM.pdf]

The genetic spectrum of congenital ocular motor apraxia type Cogan: an observational study, continued.

Simone Schröder, Gökhan Yigit, Yun Li, Janine Altmüller, Hans-Martin Büttel, Barbara Fiedler, Christoph Kretzschmar, Peter Nürnberg, Jürgen Seeger, Valentina Serpieri, Enza Maria Valente, Bernd Wollnik, Eugen Boltshauser, Knut Brockmann

Supplemental Table 1

Molecular genetic investigations and findings in 21 patients with "congenital ocular motor apraxia type Cogan" (COMA)

Patient #	Molecular genetic test	Mutated gene, pathogenic variants	Diagnostic assignment	Reference
1	MGP including *AHI1, ARL13B, ATXN10, B9D1, B9D2, C5ORF42, CC2D2A, CEP290, CEP41, CSPP1, EXOC8, GLI3, HYLS1, IFT88, INPP5E, KIAA0586, KIF7, KIF14, MKS1, NPHP1, NPHP3, OFD1, PDE6D, PDPR, RPGRIP1L, TCTN1, TCTN2, TCTN3, TMEM107, TMEM138, TMEM216, TMEM231, TMEM237, TMEM67, TTC21B, ZNF423*	*MKS1* comp. het., c.1115_1117delCCT (p.Ser372del), c.1476T>G (p.Cys492Trp)	JBTS28	
2	MGP including *AHI1, CC2D2A, CEP290, NPHP1, RPGRIP1L, TMEM216, TMEM67*	*CC2D2A* comp. het., c.3289delG (p.Val1097Phefs*2) (possible splice change), c.4583G>A (p.Arg1528His)	JBTS9	

3	ES	no conclusive result	“COMA“	
4	MGP including *AHI1, ARL13B, C5ORF42, CC2D2A, CEP164, CEP290, CEP41, CSPP1, INPP5E, KIF7, NPHP1, OFD1, PDE6D, RPGRIP1L, TCTN1, TCTN2, TCTN3, TMEM138, TMEM216, TMEM231, TMEM237, TMEM67, TTC21B, ZNF423*	*TMEM67* comp. het., c.622A>T (p.Arg208*), c.1634G>A (p.Gly545Glu)	JBTS6	
5	ES	no conclusive result	JBTS	
6	ES	*KIAA0586* comp. het., c.428delG (p.Arg143Lysfs*4), c.3888delC (p.Ile1297Serfs*19)	JBTS23	
7	Candidate gene approach: Sanger sequencing of *LAMA1*	*LAMA1* hom., c.756delT (p.Ile252Metfs*10)	PTBHS	
8*	ES	*KIAA0586* comp. het., c.428delG (p.Arg143Lysfs*4), c.1413-1G>C (splice site variant)	JBTS23	[5]

9*	ES	*KIAA0586*<br>comp. het., c.428delG (p.Arg143Lysfs*4), c.1413-1G>C (splice site variant)	JBTS23	[5]
10	ES	*SUFU*<br>het., c.83C>A (p.Ser28*)	*forme fruste* of JBTS	[6]
11	ES	*NPHP1*<br>hom. deletion of whole gene	JBTS4	
12	ES	no conclusive result	“COMA”	
13	ES	*KIAA0586*<br>comp. het., c.428delG (p.Arg143Lysfs*4), deletion of exons 8, 9 and 10	JBTS23	
14	ES	*SUFU*<br>het., c.1099G>T (p.Glu367*)	*forme fruste* of JBTS	[6]
15	ES	*NPHP1*<br>hom. deletion of whole gene	JBTS4	

16*	ES	*KIAA0586*<br>comp. het., c.428delG (p.Arg143Lysfs*4), deletion of exons 8, 9 and 10	JBTS23	
17*	ES	*KIAA0586*<br>comp. het., c.428delG (p.Arg143Lysfs*4), deletion of exons 8, 9 and 10	JBTS23	
18	ES	*ATM*<br>comp. het., c.1066-6T>G (p.?), c.2250G>A (p.Ile709_Lys750del)	variant A-T	[7]
19	ES	*SUFU*<br>het, c.479delA (p.His160Leufs*20)<br>*de novo*	*forme fruste* of JBTS	[6]
20	Candidate gene approach:<br>Sanger sequencing of *TUBA1A*	*TUBA1A*<br>het., c.82C>T (p.His28Tyr),<br>*de novo*	TUBA1A-associated brain malformation	

21	ES	*RPGRIP1L*<br>two het. variants on the same (maternal) allele, likely not causative: c.171G>T (p.Leu57Phe), c.628A>G (p.Asn210Asp)	JBTS	

Abbreviations: * = #8 and #9 as well as #16 and #17 are siblings; A-T = ataxia-telangiectasia; ES = exome sequencing; JBTS = Joubert syndrome; MGP = molecular genetic panel; PTBHS = Poretti-Boltshauser syndrome; het. = heterozygous; hom. = homozygous;

Adopted and modified from Wente et al., 2016, reference [2]
